# A second remarkable case of parapatry in a Tasmanian millipede genus (Diplopoda, Polydesmida, Dalodesmidae)

**DOI:** 10.3897/zookeys.930.38031

**Published:** 2020-04-28

**Authors:** Robert Mesibov

**Affiliations:** 1 West Ulverstone, Tasmania 7315, Australia Unaffiliated West Ulverstone, Tasmania Australia

**Keywords:** parapatry, *
Tasmaniosoma
*, Tasmania, Australia

## Abstract

*Tasmaniosoma armatum* Verhoeff, 1936 and *T. orientale* Mesibov, 2010 are parapatric in northeast Tasmania, Australia. The parapatric boundary is ca 50 km long and mainly follows streamlines. Three sections of the boundary were intensively sampled. Two gonopod variants of *T. orientale* also appear to be parapatric.

## Introduction

The endemic Tasmanian dalodesmid genus *Tasmaniosoma* Verhoeff, 1936 currently contains 22 species (http://www.millibase.org/aphia.php?p=taxdetails&id=892720 accessed 2019-07-03), some of which are distributed in mosaic parapatry. In a previous publication I documented a parapatric boundary ca 230 km long between *T. compitale* Mesibov, 2010 and *T. hickmanorum* Mesibov, 2010 in northwest Tasmania (Mesibov 2011). Here I document sections of a parapatric boundary ca 50 km long in eastern Tasmania between *T. armatum* Verhoeff, 1936 and *T. orientale* Mesibov, 2010. While the *compitale*/*hickmanorum* boundary crosses numerous streams and vegetation ecotones, the well-sampled sections of the *armatum*/*orientale* boundary mainly follow streamlines. Evidence is also presented for parapatry in two *T. orientale* gonopod variants.

## Materials and methods

### Millipede species

**Figure 1.**
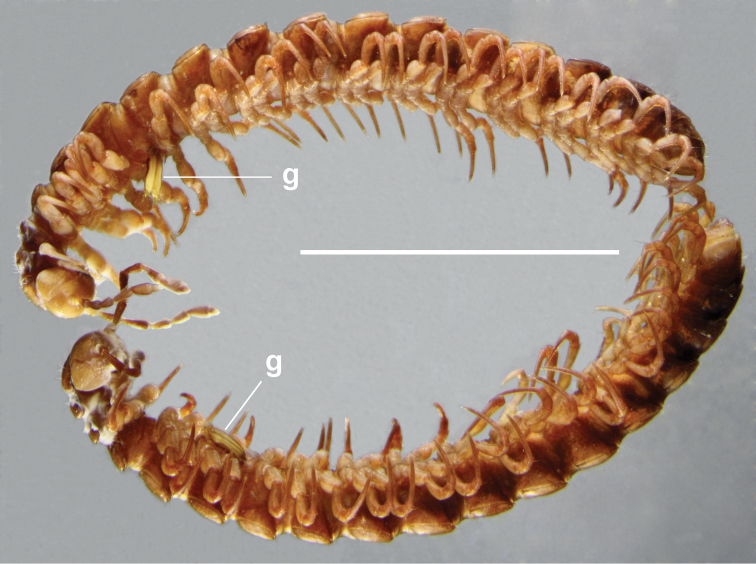
Adult males of *Tasmaniosoma armatum* Verhoeff, 1936 (top, QVM:2018:23:0093) and *T. orientale* Mesibov, 2010 (bottom, QVM:2018:23:0089). **g** indicates gonopods. The two males are from the Hop Pole Creek area (see Results section) and were collected at sites ca 0.5 km apart on the same day, 6 August 2018. Scale bar: 5 mm.

**Figure 2.**
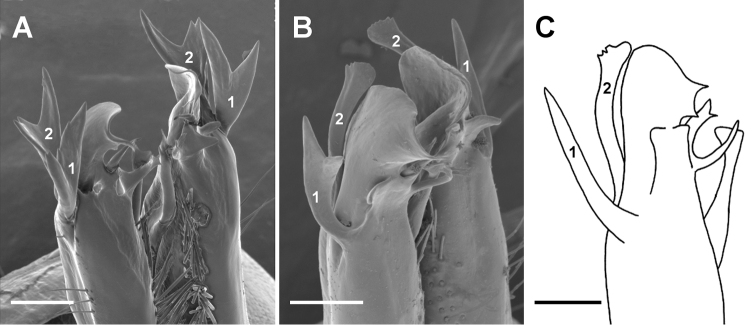
Posterolateral views of gonopod telopodite tips of *Tasmaniosoma armatum* Verhoeff, 1936 (**A**QVM:23:46567) and *T. orientale* Mesibov, 2010 (**B**QVM:23:46018 **C**QVM:23:51550, paratype). **1** = process 1, **2** = process 2. **C** shows left gonopod but is right-left reversed for easier comparison with **B**. **A–C** modified from Mesibov (2010). Scale bars: 0.1 mm.

*Tasmaniosoma armatum* and *T. orientale* are very similar in size and coloration (Fig. 1) and females of the two species are currently indistinguishable. Adult males are readily identified to species by inspection of the gonopods under low magnification (Fig. 2). On the *T. armatum* telopodite, processes 1 and 2 (Fig. 2A) are both Y-shaped, often with a small tooth inside either Y. In *T. orientale*, process 1 is Y-shaped in some populations (Fig. 2B) and simply acute in others (Fig. 2C). Process 2 in *T. orientale* is never Y-shaped but varies in length and in the shape of the apex. In most populations process 2 is gently curved medially and has a slightly expanded, flattened apex with a few small marginal teeth (Fig. 2B, C).

**Figure 3.**
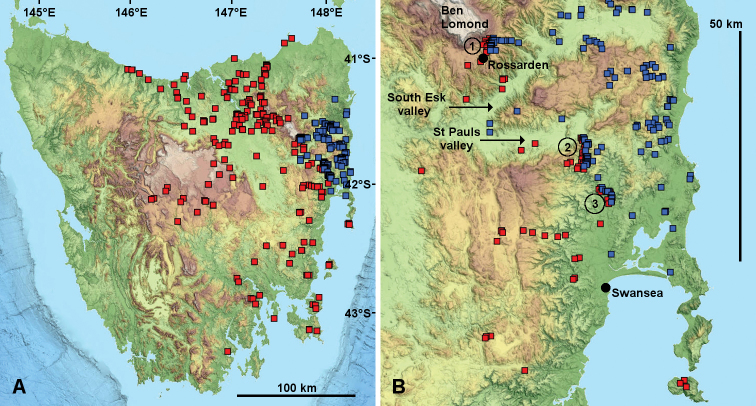
Known localities for *Tasmaniosoma armatum* Verhoeff, 1936 (red squares) and *T. orientale* Mesibov, 2010 (blue squares) as of 3 July 2019. **A** Overview of species ranges **B** Overview of parapatric zone. 1, 2 and 3 in **B** are the three areas shown in closer view in following figures.

*Tasmaniosoma armatum* occurs over ca 25000 km^2^ on Tasmania’s main island from sea level to ca 1100 m but is absent from both the western third and the northeast corner of the island (Fig. 3A). *Tasmaniosoma orientale* is restricted to ca 3000 km^2^ in the northern portion of Tasmania’s East Coast region (Fig. 3A), where it is found from sea level to at least 1000 m. Both millipede species are more abundant in open eucalypt forest and woodland than in wet eucalypt forest with a dense understory. Adults can be collected at any time of year during wet weather, but are more easily found in the cooler months (May to September). Both species wander at night as adults. During the day they shelter in loose-structured leaf, bark, and woody litter, under loose bark on standing trees, and occasionally under stones. When sheltering, *Tasmaniosoma* species rest full-length (not coiled) on damp surfaces largely free of dirt and fungal growth.

## Millipede sampling

I searched for *Tasmaniosoma* spp. on 76 field days from 2012 to 2019 at 335 sites in the western portion of the *T. orientale* range (Fig. 3B). Another 17 sites on five field days were sampled by W. and L. Clarkson in 2012 and 2013. Searches were concentrated in the neighbourhood of the presumed parapatric boundary as its location became more apparent.

Most of the searching was done in the days immediately following rainy weather. In wet periods I sometimes found several adult *Tasmaniosoma* within the first 5–10 minutes at a site. During the prolonged dry periods of the last three years of the sampling (2017–2019), I often searched a suitable site for an hour or more without success.

Millipedes were usually collected live in the field in small, screw-capped, plastic vials loosely packed with damp paper or bark fragments. Specimens were later identified, preserved in 80% ethanol and deposited as registered lots (one lot per species per collection site) in the Queen Victoria Museum and Art Gallery, Launceston, Tasmania (QVM).

Most search sites were located in the field with a Garmin Etrex 10 GPS and the locations later checked by reference to aerial photography or satellite imagery on the LISTmap website (https://maps.thelist.tas.gov.au/listmap/app/list/map). The smallest coordinate uncertainty recorded for all sites was ±25 m, to allow for the area searched around the GPS latitude/longitude. For sites yielding only a single specimen, however, the uncertainty was closer to the GPS uncertainty, ca 15 m.

### Locality data and graphics

Locality records for *T. armatum*, *T. clarksonorum*, *T. orientale* and unidentified *T. armatum*/*orientale* to 3 July 2019 are in Supplement 1 with the Darwin Core fields institutionCode, catalogNumber, phylum, class, order, family, genus, specificEpithet, scientificName, typeStatus, organismRemarks, identifiedBy, identificationRemarks, locality, country, stateProvince, decimalLatitude, decimalLongitude, geodeticDatum, coordinateUncertaintyInMeters, georeferenceSources, georeferencedBy, verbatimCoordinates, verbatimSRS, minimumElevationInMeters, maximumElevationInMeters, recordedBy, eventDate and eventRemarks.

The locality maps were generated using LISTmap tools from *theLIST* (https://maps.thelist.tas.gov.au/listmap/app/list/map), State of Tasmania. The background images (topographic maps, hillshaded maps and aerial photographs) are LISTmap background layers and the plotted points are from the author’s KML files, imported into LISTmap as external services. Habitat photographs are by the author.

## Results

### Overview

The *T. armatum* and *T. orientale* distributions meet in a zone ca 50 km long running southeast from the Ben Lomond area at ca 700 m a.s.l. to the lower Swan River valley north of Swansea at ca 30 m a.s.l. (Fig. 3B). Enough native forest and woodland remains in three portions of the zone to allow fine-scale *Tasmaniosoma* mapping: (1) near Rossarden, (2) at the northern end of the Old Coach Road, and (3) near the West Swan River/Swan River junction (areas numbered 1, 2 and 3 in Fig. 3B). Distributions in these three areas are reported separately below.

Clearing of native vegetation for farms in the South Esk River, St Pauls River and lower Swan River valleys has largely eliminated *Tasmaniosoma* populations on the wider river flats. The present-day distributions (Fig. 3B) indicate that in pre-European times the zone may have followed the South Esk downstream to its junction with the St Pauls River, then followed the latter river upstream.

I did not find *T. armatum* and *T. orientale* together at the same site anywhere in the area searched. Males with “anomalous” gonopods were collected at two sites; these are discussed below.

### Near Rossarden

**Figure 4.**
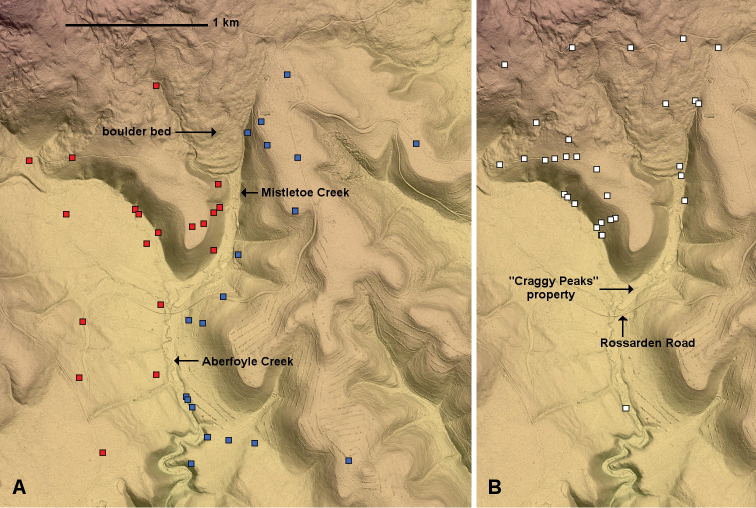
Known localities for *Tasmaniosoma* spp. near Rossarden as of 3 July 2019, on hillshaded terrain map. **A***Tasmaniosoma armatum* Verhoeff, 1936 (red squares) and *T. orientale* Mesibov, 2010 (blue squares) **B***T. clarksonorum* Mesibov, 2010 (white squares).

In dry eucalypt forest near Rossarden, *T. armatum* and *T. orientale* are parapatric along Aberfoyle Creek upstream to its junction with Mistletoe Creek (Fig. 4A). The parapatric boundary follows Mistletoe Creek upstream to ca 700 m a.s.l. in wet eucalypt forest, where the two *Tasmaniosoma* species are either absent or at very low abundance.

Mistletoe Creek is divided in the upper end of the parapatric zone into an ephemeral eastern branch and a perennial western branch. Between the two branches is a deposit of rocky rubble (“boulder bed” in Fig. 4A), lightly forested and up to ca 3 m deep. I did not find either *T. armatum* or *T. orientale* on this deposit, although *T. clarksonorum* and other native millipede species were present there in surface litter.

*Tasmaniosoma clarksonorum* Mesibov, 2010 is the dominant *Tasmaniosoma* species in wet eucalypt forest and rainforest at higher elevations in northeast Tasmania (Mesibov 2010). It is abundant at the northern, high-elevation end of the parapatric zone (Fig. 4B). *Tasmaniosoma clarksonorum* co-occurs with *T. armatum* and *T. orientale* in the riparian zones of both Aberfoyle and Mistletoe Creeks down to ca 550 m elevation.

The south-facing hillslopes south of the Rossarden Road (Fig. 4B) to the South Esk River flat have been frequently burned, and *Tasmaniosoma* spp. are very hard to find in the dry eucalypt forest on these slopes. I found scattered *T. armatum* populations east of the lower portion of Aberfoyle Creek near the South Esk River flat, which may mean that the parapatric zone in that area (if it exists) is also further east.

**Figure 5.**
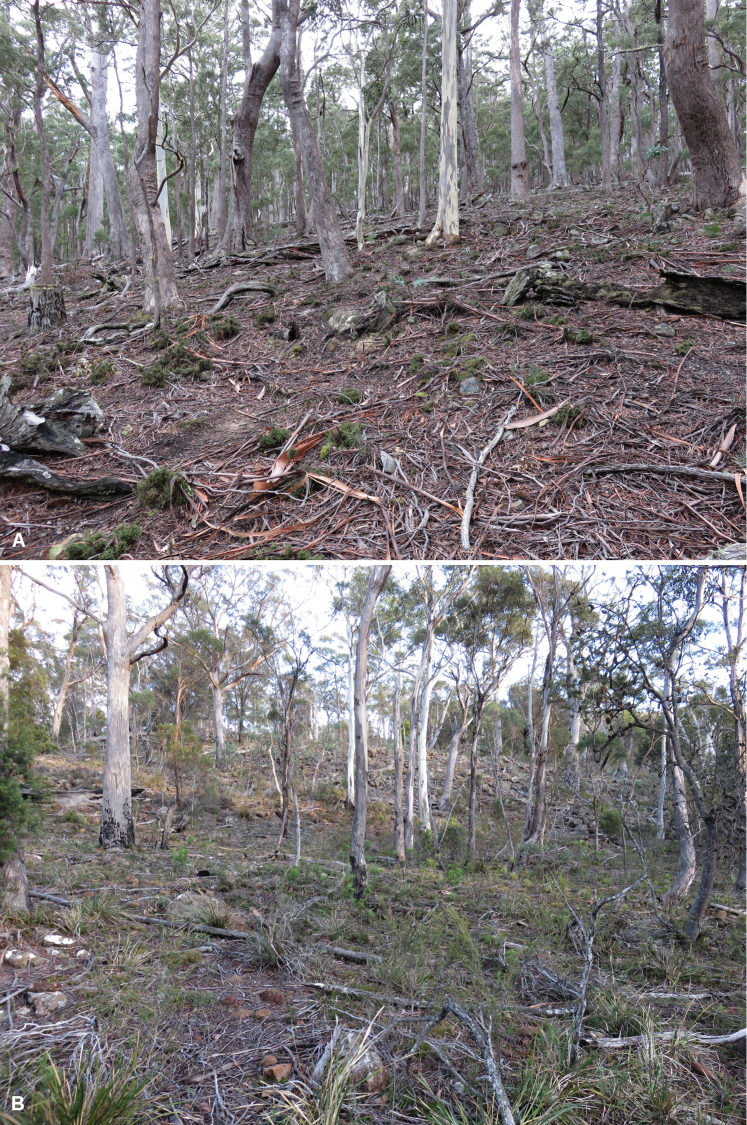
Views of dry eucalypt forest habitat. **A** Slope east of Mistletoe Creek (see Fig. 4), 12 June 2019 **B** Slope west of Hop Pole Creek (see Fig. 6), 2 July 2019.

The parapatric zone north of the Rossarden Road is on the “Craggy Peaks” private property (Fig. 4B), which in 2019 is a holiday resort with self-contained cabins. The Mistletoe Creek flat and part of the Aberfoyle Creek flat on “Craggy Peaks” were developed in the 20^th^ century for a small golf course (labelled “Ben Lomond Golf Course” on some maps). The dry eucalypt forest on the hills sloping down to the grassy flats is in good condition (Fig. 5A) and supports a diverse native invertebrate fauna.

### Northern end of the Old Coach Road

**Figure 6.**
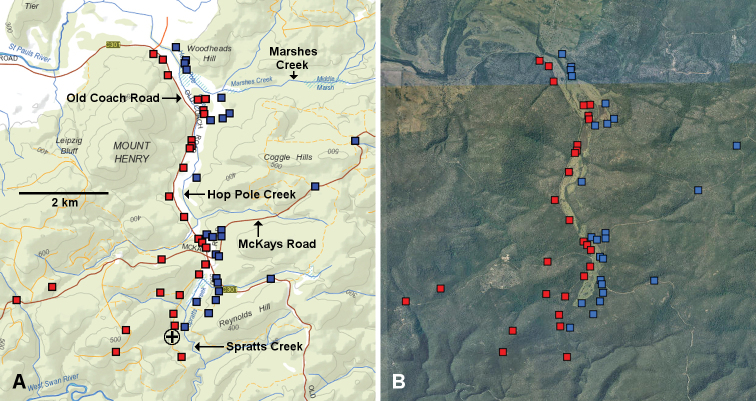
Known localities for *Tasmaniosoma armatum* Verhoeff, 1936 (red squares) and *T. orientale* Mesibov, 2010 (blue squares) at the northern end of the Old Coach Road as of 3 July 2019. **A** Topographic map with named features **B** Aerial photograph taken 25 February 2010. Circled black cross in **A** marks locality of *Tasmaniosoma* male with “anomalous” gonopods (QVM:2017:23:0173).

At the eastern end of the St Pauls River valley (Fig. 3B), the parapatric zone follows Hop Pole Creek and Marshes Creek upstream to the low watershed crossed by McKays Road (Fig. 6). From here the zone descends along Spratts Creek towards the junction with the West Swan River.

The Hop Pole Creek/Marshes Creek flat (= “Hop Pole Bottom” on some maps) and the upper portion of the Spratts Creek flat were cleared for farming in the 19^th^ century and are privately owned. Despite many years of grazing by sheep, the surrounding low, rocky hills carry dry eucalypt forest in fairly good condition (Fig. 5B).

South of the Spratts Creek flat, the creek descends towards the West Swan River in a deep, narrow valley on Crown land. The deeper parts of the valley carry denser, somewhat wetter eucalypt forest and have not been sampled.

### Near the West Swan River/Swan River junction

**Figure 7.**
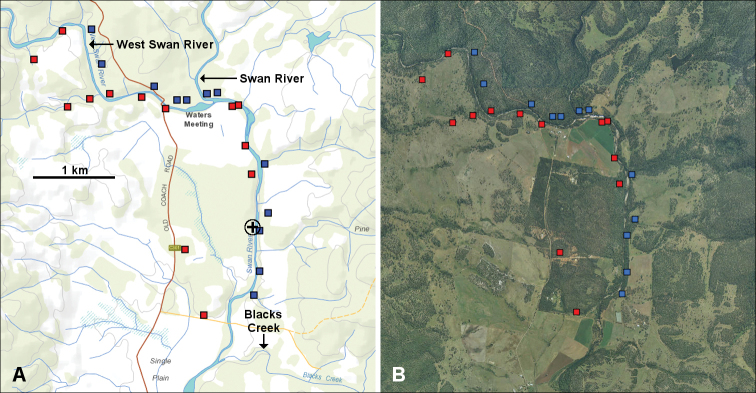
Known localities for *Tasmaniosoma armatum* Verhoeff, 1936 (red squares) and *T. orientale* Mesibov, 2010 (blue squares) near the West Swan River/Swan River junction as of 3 July 2019. **A** Topographic map with named features **B** Aerial photograph taken 1 December 2006. Circled black cross in **A** marks locality of *Tasmaniosoma* male with “anomalous” gonopods (QVM:23:54570).

*Tasmaniosoma armatum* and *T. orientale* are separated by the Swan River for at least 3 km below its junction with the West Swan River (Fig. 7). Very little native vegetation remains in the Swan River valley below the junction and close to the river. I found a few specimens of *Tasmaniosoma* spp. in riparian and near-riparian remnants below the West Swan River/Swan River junction, but none downstream from the Blacks Creek junction with the Swan.

**Figure 8.**
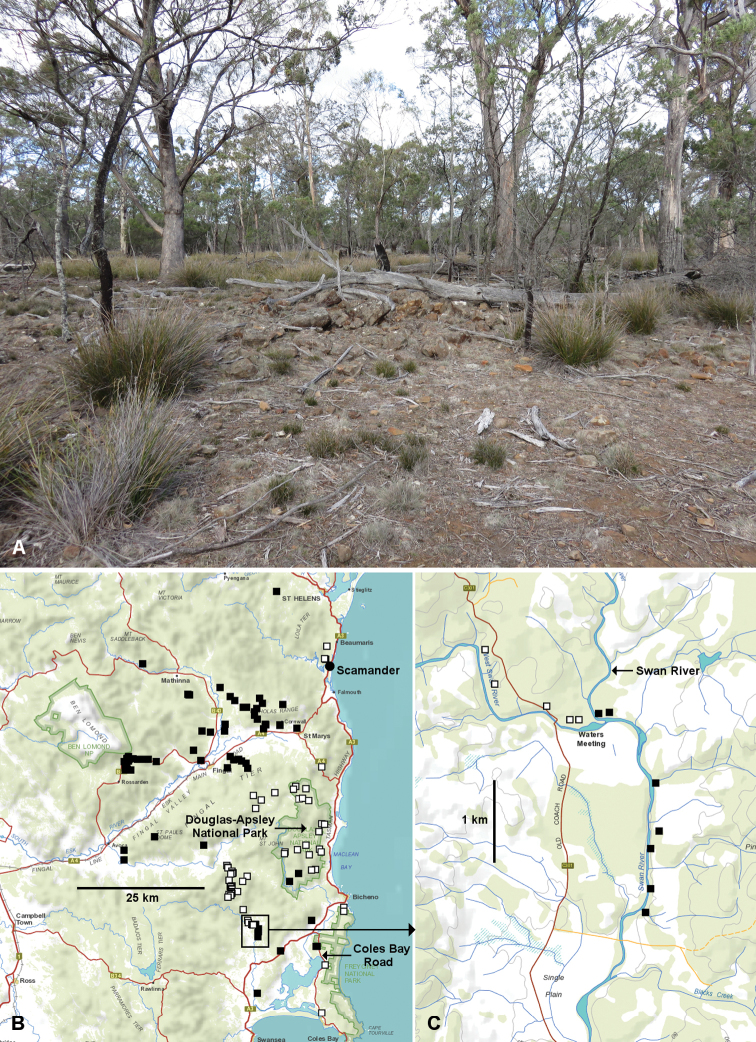
**A** View of dry eucalypt forest habitat on the north bank of the West Swan River near the West Swan River/Swan River junction (see Fig. 7), 1 July 2019 **B, C** Distributions of “Y-shaped” (black squares) and “simply acute” (white squares) process 1 variants of *Tasmaniosoma orientale* Mesibov, 2010 as of 3 July 2019 **B** Overview of *T. orientale* range **C** Near the West Swan River/Swan River junction.

For at least 3 km above the junction, *T. armatum* and *T. orientale* are mainly separated by the West Swan River. In 2017, however, I collected a male *T. armatum* on the north bank, i.e. on the *T. orientale* side of the river (Fig. 7). Millipedes are very hard to find on the north bank, which carries sparse dry eucalypt forest on stony ground (Fig. 8A).

This section of the parapatric zone is entirely on an assortment of private property blocks, with clearing for farms on the blocks beginning in the first half of the 19^th^ century.

### Males with “anomalous” gonopods

On gonopod morphology I was readily able to assign 325 males to *T. armatum* and 204 males to *T. orientale*. Two other males had a Y-shaped process 2 but a simply acute process 1 (QVM:23:54570) and a Y-shaped process 1 and a somewhat spear-shaped process 2 (QVM:2017:23:0173) (see also Fig. 2). Both were found in the parapatric zone (circled black crosses in Figs 6A, 7A) and may be hybrids.

### Gonopod variants of *T. orientale*

The geographical distributions of Y-shaped and simply acute variants of gonopod process 1 in *T. orientale* are shown in Figure 8B, C. The two distributions are largely discrete, and there are several areas in the *T. orientale* range where the variants have been collected near each other and may be parapatric: near Scamander, in the Douglas-Apsley National Park, along the Coles Bay Road and at the junction of the West Swan and Swan rivers. In the latter case the two variants appear to be separated by the Swan River (Fig. 8C).

## Discussion

### *Tasmaniosoma armatum*/*T. orientale* parapatry

I documented parapatry in *T. compitale* and *T. hickmanorum* (Mesibov 2011) as a knowledge base for future studies of millipede parapatry and speciation. The mapping of the *T. armatum* and *T. orientale* parapatric zone had a similar aim but was not as successful. Most of the native forest and woodland has long been cleared for farming in what might have been the parapatric zone in pre-European times, eliminating *Tasmaniosoma* populations. The mapping study was also limited by unfavourably dry weather during six of the seven sampling years, 2012–2019, and especially in the last three.

However, the maps presented here show that *armatum*/*orientale* parapatry in northeast Tasmania differs in one important respect from *compitale*/*hickmanorum* parapatry in northwest Tasmania. The northwest parapatric zone crosses numerous streams (Mesibov 2011: fig. 7B), while the northeast parapatric zone mainly follows streamlines. In northwest Tasmania there are numerous patches of continuous native forest within which *T. compitale* and *T. hickmanorum* are relatively abundant, and within which the parapatric zone can be crossed with a sampling transect. I found no such patches along the *armatum*/*orientale* boundary, although it is possible one exists in the lower Spratts Creek catchment (Fig. 6).

### *T. orientale* gonopod variants

Gonopod variation in *T. orientale* may represent an ongoing lineage split or splits that will eventually result in two or more reproductively isolated species. The splitting may be occurring at more than one location in the *T. orientale* range, to judge from the somewhat complicated map of variant distributions (Fig. 8B). Genetic evidence is needed in future to determine whether the variants are already isolated in areas of close parapatry.

### Future work

Besides the difference between the northwest and northeast *Tasmaniosoma* parapatric zones with respect to streamlines, three groups of more fundamental questions remain to be answered in each case: how is the parapatric boundary maintained; how, when and where did the parapatry originate; and how and when did the boundary arrive at its present position in the landscape?

Unfortunately, none of these questions can be answered from mapping evidence alone, as presented here. I have now retired from millipede studies, but I encourage other zoologists to study with genetic methods the tight parapatry documented in *Tasmaniosoma* and a number of other well-mapped Tasmanian polydesmidan genera, including *Atrophotergum* Mesibov, 2004 (Mesibov 2004), *Dasystigma* Mesibov, 2003 (Mesibov 2003a), *Gasterogramma* Jeekel, 1982 (Mesibov 2003b), and *Lissodesmus* Chamberlin, 1920 (Mesibov 2006). Much of the Tasmanian landmass still carries native vegetation in good condition and the native litter fauna (especially of millipedes) is remarkably diverse. The island is a natural laboratory awaiting investigators interested in millipede biogeography on a fine scale.

## Supplementary materials

Supplementary material 1 Specimen data for *Tasmaniosoma armatum* Verhoeff, 1936, *T. clarksonorum* Mesibov, 2010, *T. orientale* Mesibov, 2010 and specimens not yet identifiable as *T. armatum* or *T. orientale* Data type Occurrence Explanation note Data file Tasmaniosoma_specimen_data_2019-07-03.tsv. The file is a tab-separated table in UTF-8 encoding with the following Darwin Core fields: institutionCode, catalogNumber, phylum, class, order, family, genus, specificEpithet, scientificName, typeStatus, organismRemarks, identifiedBy, identificationRemarks, locality, country, stateProvince, decimalLatitude, decimalLongitude, geodeticDatum, coordinateUncertaintyInMeters, georeferenceSources, georeferencedBy, verbatimCoordinates, verbatimSRS, minimumElevationInMeters, maximumElevationInMeters, recordedBy, eventDate and eventRemarks. https://binary.pensoft.net/file/404841This dataset is made available under the Open Database License (http://opendatacommons.org/licenses/odbl/1.0/). The Open Database License (ODbL) is a license agreement intended to allow users to freely share, modify, and use this Dataset while maintaining this same freedom for others, provided that the original source and author(s) are credited.Robert Mesibov
